# Alpha-PET for Prostate Cancer: Preclinical investigation using ^149^Tb-PSMA-617

**DOI:** 10.1038/s41598-019-54150-w

**Published:** 2019-11-28

**Authors:** Christoph A. Umbricht, Ulli Köster, Peter Bernhardt, Nadezda Gracheva, Karl Johnston, Roger Schibli, Nicholas P. van der Meulen, Cristina Müller

**Affiliations:** 1 Center for Radiopharmaceutical Sciences ETH-PSI-USZ, Paul Scherrer Institute, 5232 Villigen-PSI, Switzerland; 2 Institut Laue-Langevin, 38042 Grenoble, France; 3 Department of Radiation Physics, Institution of Clinical Science, Sahlgrenska Academy, University of Gothenburg, 413 45 Gothenburg, Sweden; 4 Medical Bioengeneering, Sahlgrenska University Hospital, 413 45 Gothenburg, Sweden; 5 CERN, 1211 Geneva 23, Switzerland; 6 Department of Chemistry and Applied Biosciences, ETH Zurich, 8093 Zurich, Switzerland; 7 Laboratory of Radiochemistry, Paul Scherrer Institute, 5232 Villigen-PSI, Switzerland

**Keywords:** Cancer, Prostate

## Abstract

In this study, it was aimed to investigate ^149^Tb-PSMA-617 for targeted α-therapy (TAT) using a mouse model of prostate-specific membrane antigen (PSMA)-expressing prostate cancer. ^149^Tb-PSMA-617 was prepared with >98% radiochemical purity (6 MBq/nmol) for the treatment of mice with PSMA-positive PC-3 PIP tumors. ^149^Tb-PSMA-617 was applied at 1 × 6 MBq (Day 0) or 2 × 3 MBq (Day 0 & Day 1 or Day 0 & Day 3) and the mice were monitored over time until they had reached a pre-defined endpoint which required euthanasia. The tumor growth was significantly delayed in mice of the treated groups as compared to untreated controls (p < 0.05). TAT was most effective in mice injected with 2 × 3 MBq (Day 0 & 1) resulting in a median lifetime of 36 days, whereas in untreated mice, the median lifetime was only 20 days. Due to the β^+^-emission of ^149^Tb, tumor localization was feasible using PET/CT after injection of ^149^Tb-PSMA-617 (5 MBq). The PET images confirmed the selective accumulation of ^149^Tb-PSMA-617 in PC-3 PIP tumor xenografts. The unique characteristics of ^149^Tb for TAT make this radionuclide of particular interest for future clinical translation, thereby, potentially enabling PET-based imaging to monitor the radioligand’s tissue distribution.

## Introduction

In recent years, targeted radioligand therapy has emerged as a promising option for patients suffering from metastatic castration-resistant prostate cancer (mCRPC)^1^. PSMA-617 is a small-molecular-weight ligand used to target the prostate-specific membrane antigen (PSMA), which is overexpressed in most prostate cancer cases^2^. It has been used in combination with ^177^Lu, a β^−^-particle-emitting radiolanthanide, for the treatment of mCRPC patients^3^. In the majority of treated patients, the tumor lesions and PSA levels were reduced after multiple cycles of ^177^Lu-PSMA-617 therapy^4^, however, complete remission remained rare and some patients still showed progressive disease after several therapy cycles^3,5^. Targeted α-therapy (TAT) has, therefore, been proposed due to the known increased radiobiological effectiveness of α-particles as compared to β^−^-particles^6^.

**Figure 1:**
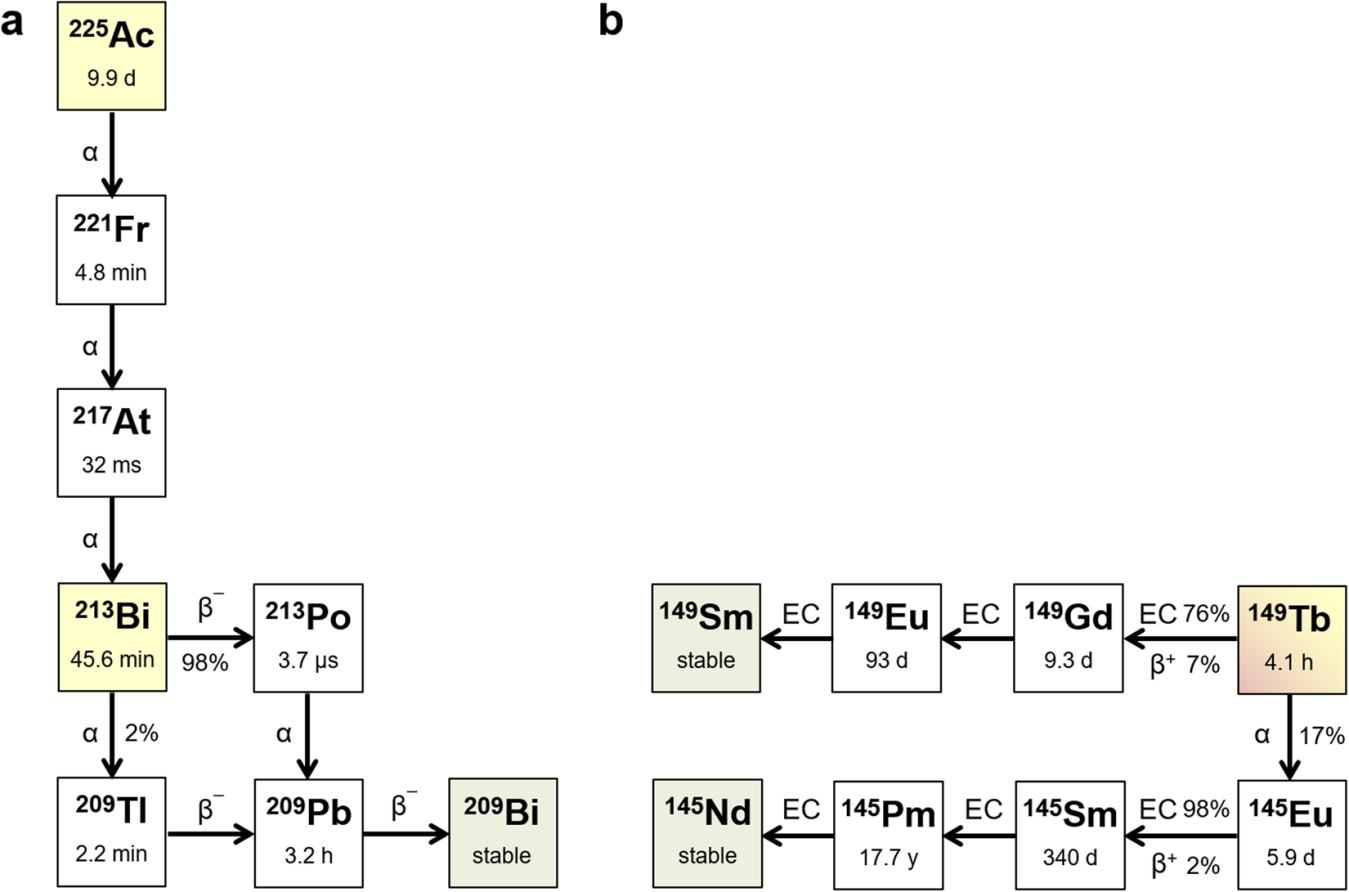
Principle decay schemes of ^225^Ac, ^213^Bi and ^149^Tb. (**a**) Decay scheme of ^225^Ac and ^213^Bi. (**b**) Decay scheme of ^149^Tb.

First-in-man studies using α-emitters, such as ^225^Ac and ^213^Bi, were performed in patients with ^177^Lu-resistant disease or when ^177^Lu-PSMA-617 was contra-indicated, due to excessive involvement of bone lesions and the inherent risk of bone marrow toxicity as a consequence of ^177^Lu-PSMA-617 accumulation^7,8^. The results of ^225^Ac-PSMA-617 therapy were impressive and illustrated the efficacy of α-emitters to kill cancer cells that had become resistant to more conventional therapies. ^213^Bi-PSMA-617 showed remarkable effects in a mCRPC patient who was determined to be progressive using conventional therapy^8^. Undoubtedly, α-therapy has the potential to be effective in patients with metastasized cancer, however, both ^225^Ac- and ^213^Bi-based radioligands are associated with currently unsolved challenges regarding the therapeutic window and logistics. ^225^Ac, with a relatively long half-life of 9.9 d, decays via several α- and β^−^-disintegrations through its daughters to ^209^Bi (Fig. 1a)^9,10^. Since the radiometal is released from the chelator during the first α-decay^11^, the subsequent decay of daughter nuclides may occur at sites in the body other than the tumor lesions, potentially causing undesired side effects. The decay scheme of ^213^Bi may be of less concern in view of toxicity to healthy tissue, however, its short half-life of only 46 min makes it generally unsuitable for systemic therapy (Fig. 1a).

Herein, we propose ^149^Tb as a potential alternative α-emitter for targeted radioligand therapy, based on several attractive features: (i) ^149^Tb decays with a half-life of 4.1 h, which is relatively short as compared to ^225^Ac, but more than four-fold longer than the half-life of ^213^Bi. This situation makes ^149^Tb particularly interesting in combination with small molecules that are characterized by fast accumulation in the tumor lesions and efficient clearance from healthy tissue. (ii) ^149^Tb emits low-energy α-particles (E_α_ = 3.97 MeV; I = 17%), but the decay does not involve relevant α-emitting daughter nuclides, which is advantageous over ^213^Bi and ^225^Ac (Fig. 1b). (iii) The co-emission of β^+^-particles (positrons) is a unique feature of ^149^Tb, making it suitable to trace ^149^Tb-labeled radioligands using positron emission tomography (PET). This has recently been exemplified in a preclinical pilot study, in which we demonstrated the feasibility of visualizing ^149^Tb using PET and referred to this approach as “alpha-PET^12^”. (iv) ^149^Tb, as a radiolanthanide, can be stably coordinated with a DOTA chelator and, hence, be used with any established tumor-targeting agent that is also applied for ^177^Lu-therapy. (v) Finally, it is important to recognize that additional, medically-interesting Tb radioisotopes exist, among those ^161^Tb, which has similar characteristics to ^177^Lu but co-emits conversion and Auger electrons that were shown to potentiate the therapeutic efficacy in a preclinical setting^13–16^. This situation could enable using chemically-identical radioligands for either β^−^-/Auger electron therapy or TAT, respectively.

The potential of ^149^Tb was demonstrated for the first time in a preclinical therapy study more than a decade ago^17^. It was shown that ^149^Tb-rituximab was able to specifically kill circulating cancer cells and small cell clusters in a leukemia mouse model. The therapeutic efficacy of ^149^Tb was also investigated by our own group using a ^149^Tb-labeled DOTA-folate conjugate in a therapy study with KB tumor-bearing mice^12^.

In this study, ^149^Tb was used for the labeling of PSMA-617 and tested in a preclinical setting. ^149^Tb-PSMA-617 was investigated in a therapy experiment with tumor-bearing mice using variable application schemes and for the visualization of PSMA-positive tumor xenografts using preclinical PET/CT.

## Results

### Preparation of ^149^Tb-PSMA-617

Directly after separation from zinc and isobar impurities, the final product (^149^Tb in HCl 0.05 M) was used for the labeling of PSMA-617. ^149^Tb-PSMA-617 was obtained at a molar activity of up to 6 MBq/nmol, with a radiochemical purity of >98%. The retention time of the product (t_R_ = 8.7 min) was equivalent to previous data obtained with ^177^Lu-PSMA-617 (Supplementary Information, Fig. S1)^18^.

### Areas under the curve (AUC) and AUC ratios of ^149^Tb-PSMA-617

Based on previous studies that showed equal distribution of ^177^Lu- and ^161/152^Tb-labeled tumor targeting agents (including DOTA-folate^14^, DOTANOC^19^ and PSMA-617^16^), it was assumed that ^149^Tb-PSMA-617 and ^177^Lu-PSMA-617 would distribute equally in the body. The distribution of ^177^Lu-PSMA-617 showed fast accumulation in PC-3 PIP tumor xenografts, with the kidneys being the only healthy organs with substantial accumulation of activity (Supplementary Information, Table S1)^2^. The biodistribution data obtained with ^177^Lu-PSMA-617 were transformed to non-decay-corrected data, using the half-life of ^149^Tb, to obtain the time-dependent uptake of ^149^Tb-PSMA-617 in the various tissues. This enabled the determination of the areas under the curves (AUCs) and the respective tumor-to-background AUC ratios. Due to the much shorter half-life of ^149^Tb as compared to ^177^Lu, the activity retention in the tumor xenograft was shorter resulting in low uptake values (0.66 ± 0.10% IA/g) at 24 h p.i. In any normal tissue and organ the retention of activity was <0.1% IA/g at 24 h after injection of ^149^Tb-PSMA-617 (Supplementary Information, Table S2). The tumor-to-blood, tumor-to-kidney and tumor-to-liver AUC ratios of ^149^Tb-PSMA-617 were determined as 74, 10 and 225, respectively (Table 1). Based on the pharmacokinetic properties of radiolabeled PSMA-617, characterized by high retention of tumor-accumulated activity but fast excretion from background organs, the AUC ratios correlated positively with the half-life of the respective radionuclide. Calculations of AUC ratios for ^213^Bi-PSMA-617 - under the assumption that it would distribute the same as ^177^Lu-PSMA-617 - revealed clearly lower values than determined for ^149^Tb-PSMA-617 (Table 1). Accordingly, calculated AUC ratios of ^225^Ac-PSMA-617 resembled more closely those of ^177^Lu-PSMA-617. It has to be critically acknowledged, however, that the daughter nuclides and their uncontrollable decay, also potentially in non-targeted tissues, has not been taken into consideration for this estimation.

**Table 1:** Area under the curve calculations for tumor, blood and kidney after injection of ^177^Lu-PSMA-617, ^149^Tb-PSMA-617, ^213^Bi-PSMA-617 and ^225^Ac-PSMA-617.

0 −∞ h p.i.	Experimental Data*	Theoretical Values (calculated based on half-life)		
AUC Organ	^177^Lu-PSMA-617	^149^Tb-PSMA-617	^213^Bi-PSMA-617	^225^Ac-PSMA-617
Tumor	4050	358	47	4703
Blood	6.0	4.86	5	6.1
Kidney	51	34	22	52
Liver	8.1	1.6	1.1	11
**AUC Ratios**	^**177**^**Lu-PSMA-617**	^**149**^**Tb-PSMA-617**	^**213**^**Bi-PSMA-617**	^**225**^**Ac-PSMA-617**
Tumor-to-blood	673	74	9.4	771
Tumor-to-kidney	79	10	2.2	91
Tumor-to-liver	502	225	44	414

The calculations are based on experimental data acquired with ^177^Lu-PSMA-617^32^, under the assumption that the radioligands would distribute equally irrespective of the coordinated radionuclide.

*Data are based on previously-published data in Benešová *et al*.^32^. Data reused with permission from (Benešová et al. 2018 Mol Pharm 15(3):934-946). Copyright (2019) American Chemical Society.

### Dose estimation for ^149^Tb-PSMA-617 and ^177^Lu-PSMA-617

The calculated mean specific absorbed doses of ^149^Tb-PSMA-617 to the tumor and kidneys determined values of 1.5 Gy/MBq and 0.14 Gy/MBq, respectively. Due to the increased radiobiological effectiveness (RBE) of α-particles as compared to β^−^-particles^20^, the estimated equivalent dose to tumors and kidneys was calculated at 6.9 Sv_RBE5_/MBq and 0.63 Sv_RBE5_/MBq, respectively, when using ^149^Tb-PSMA-617.

The calculations for ^177^Lu-PSMA-617 were performed in analogy and revealed a mean specific absorbed dose to the tumors and kidneys of 3.2 Gy/MBq and 0.041 Gy/MBq, respectively (Supplementary Information). It should be noted that these dosimetry estimations are based on the assumptions of an average sphere size of 60 mm^3^, while interindividual differences in tumor sizes were not considered. The variation in absorbed doses due to tumor size variations was, however, less than 5% for ^149^Tb and ^177^Lu, respectively.

### Therapy study

**Figure 2:**
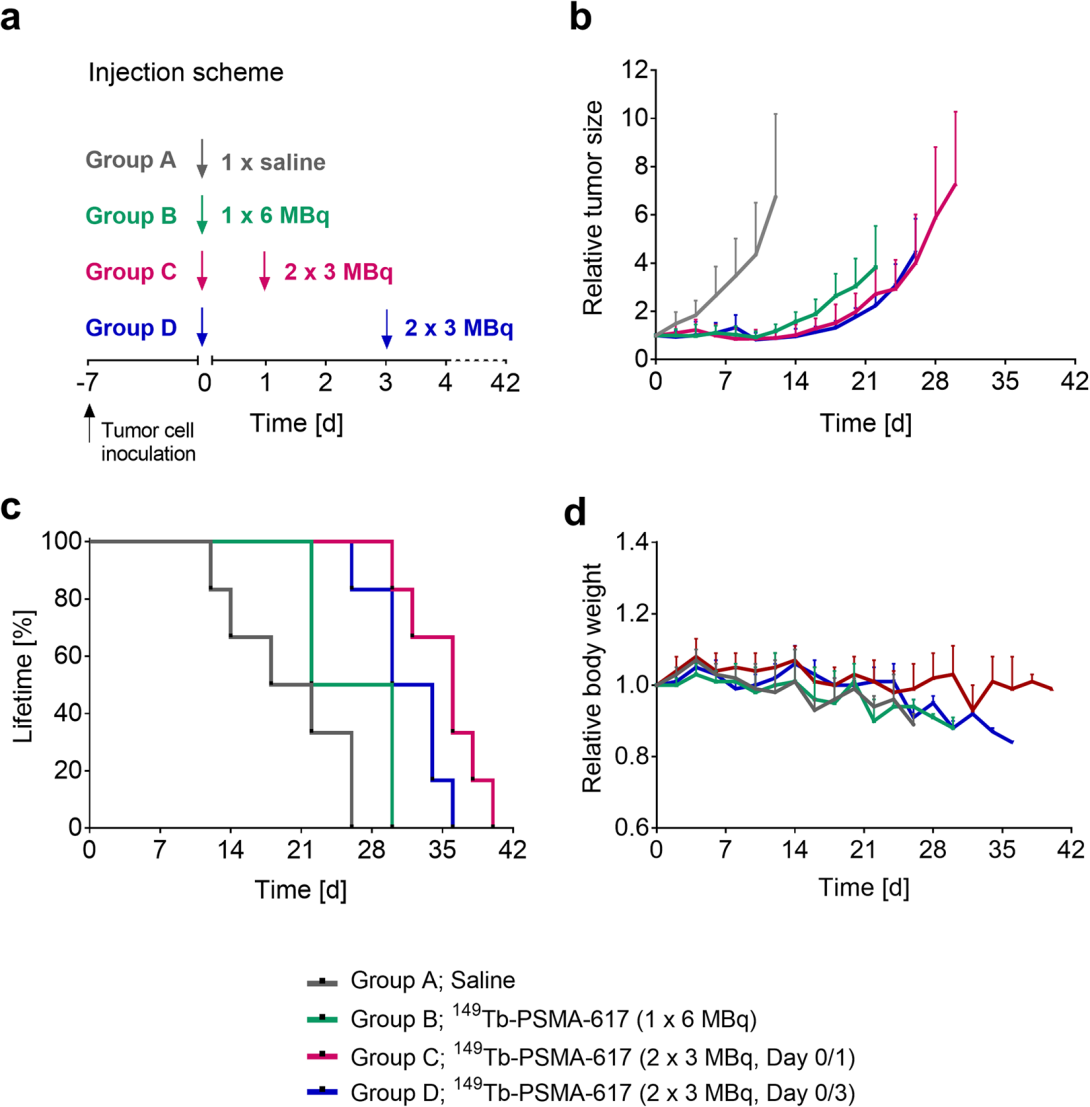
Analysis of therapy study performed with ^149^Tb-PSMA-617 in PC-3 PIP tumor-bearing mice. (**a**) Timeline of the application of ^149^Tb-PSMA-617 to the various groups of mice. (**b**) Tumor growth curves of Groups A–D relative to the tumor volume at Day 0 (set to 1). Data shown until the first mouse of the group in question reached a predefined endpoint. (**c**) Curves reflecting the lifetime of mice of Groups A–D (mice were euthanized when they reached one or several of the predefined endpoints). (**d**) Relative body weight of mice of Groups A–D.

Mice from four groups were injected with either only saline or a cumulative activity of 6 MBq ^149^Tb-PSMA-617 using variable injection schemes (Fig. 2a). Group A (control group; injected with saline) showed constant tumor growth and, as a result, the first mouse had to be euthanized at Day 12 due to an oversized tumor. Tumor growth of mice in Group B, which received one injection of 6 MBq ^149^Tb-PSMA-617 at Day 0, was clearly reduced compared to the control group. The first three mice of Group B had to be euthanized at Day 22 due to loss of body weight, presumably as a consequence of the tumor burden. The tumor growth inhibition in mice from Group C and D that received 2 × 3 MBq ^149^Tb-PSMA-617 at Day 0 and Day 1 or at Day 0 and Day 3, respectively, was comparable between the two groups. The first mouse from Groups C and D, respectively, was euthanized at Day 30 and Day 26, due to a combination of body weight loss and increased tumor volume (Fig. 2b and Table 2).

**Table 2:** Treatment group, day when the first mouse of the group had to be euthanized, median lifetime, tumor growth inhibition (TGI) and tumor growth delay index with 2- and 5-fold increase of tumor size (TGDI_2_ and TGDI_5_) of Groups B–D, respectively, as compared to the untreated control mice of Group A.

Group	Treatment Group (n = 6)	First mouse euthanized [Day]	Median^(a)^ lifetime [days]	TGI [%]	TGDI_2_	TGDI_5_
A	saline	12	20	0 ± 47	1.0 ± 0.5	1.0 ± 0.3
B	^149^Tb-PSMA-617^(b)^	22	26	82 ± 3.9*	2.7 ± 0.2*	2.0 ± 0.1*
C	^149^Tb-PSMA-617^(c)^	30	36	87 ± 4.5*	3.4 ± 0.5*	2.4 ± 0.2*
D	^149^Tb-PSMA-617^(d)^	26	32	87 ± 4.8*	3.5 ± 0.2*	2.3 ± 0.1*

Values indicated as average ± SD.

^(a)^The lifetime was based on euthanasia required, according to pre-defined endpoints.

^(b)^Injected with 6 MBq at Day 1.

^(c)^Injected with 3 MBq at Day 0 and Day 1, respectively.

^(d)^Injected with 3 MBq at Day 0 and Day 3, respectively.

*Indicating significant difference from the control group (p < 0.05).

Remark: TGDI_2_ and TGDI_5_ values differed significantly between Groups B and C.

Quantification of the therapeutic effect by means of calculating the tumor growth inhibition (TGI) revealed a significantly (p < 0.05) increased value for Groups B, C and D as compared to Group A. The same was found when calculating the tumor growth delay indices 2 and 5 (TGDI_2_ and TGDI_5_), which were significantly (p < 0.05) larger in treated mice (Groups B-D) as compared to the control group (Group A). Among the treated mice, these values were highest for mice from Groups C and D. The lifetime of mice was based on the day of euthanasia which was required according to pre-defined endpoints. When compared to the control mice (median lifetime: 20 days), the treated mice had an increased median lifetime of 26 days (Group B), 36 days (Group C) and 32 days (Group D), respectively (Fig. 2c and Table 2).

### Monitoring of mice during the therapy study

Monitoring of the mice also revealed body weight loss over time in all groups except Group C, which was observed as a consequence of increasing tumor burden (Fig. 2d). The analysis of blood plasma parameters at the time of euthanasia indicated no significant changes in any of the measured parameters between treated mice of Groups B-D and untreated control mice of Group A (Supplementary Information, Table S3). Moreover, the average body weight at the time of euthanasia, as well as the organ mass of kidneys, liver and brain, and the ratios thereof did not reveal any significant differences among the mice of the different groups (Table 3).

**Table 3:** Body weight and organ weight of mice in the therapy study and their corresponding ratios.

Group (n = 6)	Whole body weight^a^ [g] (average ± SD)	Organ weight^a^ [mg] (average ± SD)			Organ weight ratio (average ± SD)	
		Kidneys	Liver	Brain	Kidney-to-brain	Liver-to-brain
Group A	14.5 ± 0.62	184 ± 25	779 ± 138	358 ± 14	0.51 ± 0.07	2.2 ± 0.44
Group B	14.7 ± 1.02	223 ± 146	743 ± 154	361 ± 30	0.62 ± 0.40	2.1 ± 0.40
Group C	15.7 ± 1.24	195 ± 23	795 ± 77	377 ± 18	0.52 ± 0.05	2.1 ± 0.16
Group D	14.4 ± 0.42	185 ± 7	801 ± 49	366 ± 19	0.51 ± 0.04	2.2 ± 0.17

Values indicated as average ± standard deviation (SD).

^a^Data obtained at the day of euthanasia when an endpoint criterion was reached.

### PET/CT imaging studies

**Figure 3:**
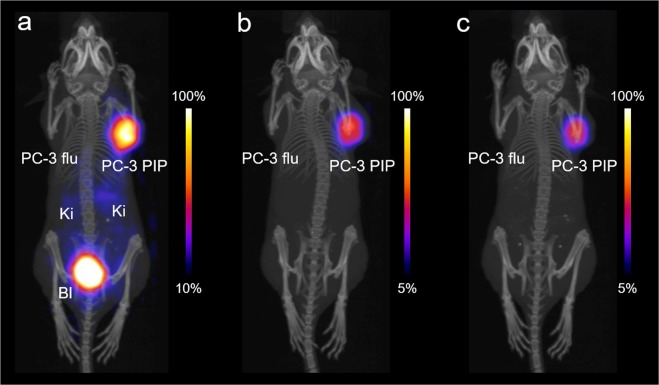
Maximum intensity projections of PET/CT scans of a mouse bearing a PSMA-positive PC-3 PIP tumor xenograft (right shoulder) and PSMA-negative PC-3 flu (left shoulder) tumor xenografts. (**a**) PET/CT scan obtained 30 min after injection of ^149^Tb-PSMA-617. (**b**) PET/CT scan obtained 2 h after injection of ^149^Tb-PSMA-617. (**c**) PET/CT scan obtained 4 h after injection of ^149^Tb-PSMA-617. PC-3 PIP = PSMA-positive tumor; PC-3 flu tumor = PSMA-negative tumor, Ki = kidneys, Bl = urinary bladder.

In a separate experiment, PET/CT scans were performed with PC-3 PIP/flu tumor-bearing mice at 30 min, 2 h and 4 h after injection of 5 MBq ^149^Tb-PSMA-617 (Fig. 3, Supplementary Information Fig. S2). Significant uptake of radioactivity was detected in the PC-3 PIP tumors (right shoulder), while accumulation of the radioligand in PC-3 flu tumors (left shoulder) was not observed. In normal tissues and organs, activity accumulation was only visible in the kidneys at early time points after injection and in the urinary bladder as a result of the renal excretion of the radioligand.

## Discussion

In the present study, ^149^Tb was produced at a quantity and quality that enabled the labeling of PSMA-617 at a specific activity and radiochemical purity suitable for a preclinical therapy study. The experiment was designed with four groups of six mice, namely, one group of untreated control animals and three groups of mice treated with ^149^Tb-PSMA-617 according to different application schemes. The treated groups of mice that received ^149^Tb-PSMA-617 in two fractions showed a somewhat better tumor growth inhibition than the group of mice that received a single application of ^149^Tb-PSMA-617. Overall, mice that received ^149^Tb-PSMA-617 on two consecutive days reached the endpoints later than mice of the other groups, as reflected by highest median lifetime and a stable body weight over the entire time of investigation.

Dosimetry estimations revealed a mean absorbed tumor dose of 6.9 Sv_RBE5_/MBq for ^149^Tb-PSMA-617, which was about double the value calculated for the tumor dose after application of ^177^Lu-PSMA-617 (3.2 Gy/MBq). Speculations regarding the required activity of ^149^Tb-PSMA-617 for a clinical application appear difficult, based on the dosimetry estimation from a preclinical setting. The TAT recently performed with ^213^Bi-PSMA-617 in a patient used a cumulative activity of ~600 MBq, applied in two cycles^8^. The outcome was an impressive molecular imaging result, as well as biochemical response, with significantly reduced PSA values after 11 months^8^. According to the decay properties of the radionuclides in question, ^149^Tb-PSMA-617 is likely to be more potent than ^213^Bi-PSMA-617 and, therefore, most probably equally effective at lower activities.

The mean absorbed dose of ^149^Tb-PSMA-617 to the kidneys was determined to be ~10-fold higher than that of ^177^Lu-PSMA-617. In a previously-performed therapy study in mice performed with ^177^Lu-folate, a dose level of ~23 Gy to the kidneys was well tolerated^21^. Should this renal dose limit be translatable to α-emitters, one could still apply 6 cycles safely with ^149^Tb-PSMA-617 (using 6 MBq per mouse with a cumulative activity of 36 MBq) resulting in accumulative dose of ~23 Sv_RBE5_ to the kidneys.

Radionephrotoxicity in patients treated with ^177^Lu-PSMA-617 has not been observed, due to the low renal uptake and, consequently, low mean aborbed dose to the kidneys (~0.6 Gy/GBq)^22,23^. It is, therefore, likely that the generally-accepted (conservative) threshold dose of ~23 Gy^24,25^ would not be reached with ^149^Tb-PSMA-617, since the quantity of injected activity would be significantly lower. It can even be expected that the renal dose would still be within the safety margins if it was increased by a factor of 10 (i.e. ~6 Sv_RBE5_/GBq), as observed in this preclinical study. Importantly, the calculated absorbed kidney dose reported for ^213^Bi-PSMA-617 in patients was determined to be in a similar range (~8 Sv_RBE5_/Gy)^26^.

Our calculations of AUC ratios from preclinical data indicated the highest and, thus, most favorable ratios for the longer-lived ^225^Ac. These results were in line with literature reports on theoretical dose estimations that considered ^225^Ac-PSMA-617 to be superior to ^213^Bi-PSMA-617, due to favorable dosimetry, with an increased therapeutic index and less off-target radiation^26^. It is, however, important to recognize that ^225^Ac decays by several α- and β^−^-disintegrations, which may add to the off-target dose. The fact that ^149^Tb does not have relevant α-emitting daughters adds particular value to this radionuclide. As the tumor-to-background AUC ratios increase with the half-life of the applied radionuclide, ^149^Tb would be a clearly more favorable α-emitter than ^213^Bi for TAT. The four-fold increased half-life of ^149^Tb, as compared to ^213^Bi would not only improve the tumor-to-background dose ratios but also facilitate the logistics of radioligand preparation and distribution. These promising circumstances warrant the evaluation of new production sites to make ^149^Tb routinely available at larger quantities.

Due to the positron emission of ^149^Tb, the accumulation of ^149^Tb-PSMA-617 in PSMA-positive prostate tumor xenografts was readily visualized using preclinical PET. This approach was previously demonstrated with ^149^Tb-DOTANOC^12^. The unique characteristic of ^149^Tb to emit α-particles and positrons (previously referred to as the concept of “alpha-PET”) would most likely allow the imaging of ^149^Tb-based α-therapy in patients. This would give ^149^Tb an advantage over existing α-emitters and provide a new dimension in view of its clinical translation. It would also allow accurate retrospective dose estimations to plan future applications and minimize off-target toxicity.

Potential limitations of this study include the fact that the PC-3 PIP tumor mouse model is based on PCa cells that were transduced to stably express PSMA at levels which are higher than in LNCaP tumor xenografts that express PSMA physiologically^27^. Moreover, tumor xenografts based on PC-3 PIP cells express PSMA homogeneously throughout the xenograft, which may not exactly reflect the situation of lesions in patients. Finally, a human xenograft only grows in immune-deficient (athymic nude) mice, hence, immunological reactions, which may have an impact on the therapy outcome, are not considered in this model.

The mice were treated when the tumor xenografts were still quite small, in order to enable monitoring of the tumor growth (delay) over a reasonable time period as commonly performed in preclinical settings^16,28,29^. This may be seen as a limitation, since tumor lesions in patients may have developed over several weeks. It is, however, important to mention that the patients suffering from metastatic disease with very small lesions would profit most from TAT. It is, thus, vital to show the therapy effect in small tumors since these smallest lesions are commonly the ones, which do not get sufficient dose when using the current generation of β^−^-emitting radionuclides such as ^177^Lu^30^.

A further limitation of any preclinical study refers to the legal requirements of defining endpoints, when mice have to be euthanized, which do not necessarily reflect the situation of a cancer patient. In this study, the endpoints of mice were defined based on the tumor size and body weight loss according to ethical guidelines of the local law of animal protection.

## Conclusion

The interesting features of ^149^Tb for “alpha-PET” make it attractive for in-depth preclinical follow-up investigations. Certainly, higher quantities of activity and/or more frequent injections of ^149^Tb-PSMA-617 would be necessary to eradicate the tumors entirely. This was, however, not feasible in this study due to the still limited availability of ^149^Tb. Beyond the application of ^149^Tb-PSMA-617, ^149^Tb could be employed in combination with a large variety of DOTA-functionalized, tumor-targeting ligands used in clinics or currently under development. A potential clinical translation of ^149^Tb-based radionuclide therapy may, thus, become a realistic future perspective, provided that a significant scale-up of the current production capabilities can be achieved by establishing effective new production sites.

## Materials and Methods

### Production and chemical separation of ^149^Tb

^149^Tb was produced by proton-induced spallation in a tantalum target, followed by ionization of the spallation products and online mass separation at the ISOLDE facility (CERN, Geneva, Switzerland), as previously reported^12,13,31^. The foils, containing the 149 isobars, were transported to PSI where the ^149^Tb was chemically separated from the zinc, as well as from the isobar and pseudo-isobar impurities using chromatographic methods. The final product was obtained as ^149^TbCl_3_ in a small volume of 0.05 M HCl, which enabled its application for direct radiolabeling. A detailed description of the separation process will be published elsewhere.

### Preparation of ^149^Tb-PSMA-617

The labeling of PSMA-617 (Advanced Biochemical Compounds, ABX GmbH, Radeberg, Germany) with ^149^Tb was performed according to a standard radiolabeling protocol at pH 4.5^18^. An aliquot of ^149^TbCl_3_/HCl (0.05 M) was added to a mixture of sodium acetate (0.5 M, pH ~8) and HCl (0.05 M) containing PSMA-617 to obtain the required molar activity of 3 MBq/nmol or 6 MBq/nmol, respectively. The reaction mixture was incubated for 15 min at 95 °C, followed by quality control using HPLC (Supplementary Information)^32^. The *in vivo* experiments were performed using ^149^Tb-PSMA-617 without further purification.

### Estimation of AUC ratios of ^149^Tb-PSMA-617

In this study, it was assumed that the tissue distribution of ^149^Tb-PSMA-617 was equal to ^177^Lu-PSMA-617, which enabled us to use previously-published biodistribution data obtained with ^177^Lu-PSMA-617^32^ with permission from (Benešová et al. 2018 Mol Pharm 15(3):934-946). Copyright (2019) American Chemical Society. Transformation of these data to non-decay-corrected data using the half-life of ^149^Tb revealed the effective uptake of ^149^Tb-PSMA-617 in the tumors, blood, kidneys and liver over time. The time-activity curves for the tumor were obtained with a mono-exponential function, while a bi-exponential function was utilized for the kidney, liver and blood, fitted to the non-decay-corrected data points using MATLAB. The time-integrated activity was obtained by integration to infinity. These AUC values were used to determine the tumor-to-blood, tumor-to-kidney and tumor-to-liver AUC ratios for ^149^Tb-PSMA-617 as a measure of the dose ratios. The data also enabled the comparison of the dose ratios with those theoretically obtained when PSMA-617 would be used in combination with other α-emitters, such as ^213^Bi (T_1/2_ = 46 min) and ^225^Ac (T_1/2_ = 9.9 d), under the assumption that the tissue distribution would be identical in this mouse model.

### Dosimetry estimations for ^149^Tb-PSMA-617 and ^177^Lu-PSMA-617

The mean specific absorbed doses (Gy/MBq) to the tumors and kidneys were calculated by multiplication of time-integrated activity concentration (corresponding to the AUC values), by the emitted α-energy (663 kev/decay) and the emitted electron energy (86 keV/decay) for ^149^Tb. The emitted photon energy, as well as the electron energy emitted from the daughter radionuclides (^149^Gd, ^145^Eu, and ^145^Sm), was omitted. The absorbed electron fractions for tumors and kidneys were assessed by Monte Carlo simulations using PENELOPE-2014^33^ and a conversion factor. Due to the increased radiobiological effectiveness (RBE) of α-particles as compared to β^−^-particles^20,34,35^, the estimated equivalent dose was calculated using a RBE of 5 for the energy emitted as α-particles (663 keV/decay) and the RBE reset to 1 for the emitted electrons (86 keV/decay); the resulting unit is indicated as Sv_RBE5_. The calculations for ^177^Lu-PSMA-617 were performed in analogy (Supplementary Information).

### *In vivo* studies

*In vivo* experiments were approved by the local veterinarian department and conducted in accordance with the Swiss law of animal protection. The preclinical studies have been ethically approved by the Cantonal Committee of Animal Experimentation and permitted by the responsible cantonal authorities (license number 75668). Athymic BALB/c nude mice were obtained from Charles River Laboratories (Sulzfeld, Germany) at the age of 5–6 weeks.

### Tumor cells

Sub-lines of the androgen-independent PC-3 human prostate cancer xenograft, originally derived from an advanced androgen-independent bone metastasis, were kindly provided by Prof. M. Pomper (Johns Hopkins University, Medical School, Baltimore, U.S.A.). The cell lines are transduced to express high levels of PSMA (PC-3 PIP) or mock-transduced as a PSMA-negative control (PC-3 flu)^27^. PC-3 PIP/flu tumor cells are widely used in the community for preclinical studies to evaluate PSMA-targeted radioligands^28,29,32,36–39^. It was previously reported that PC-3 PIP cells express PSMA at significantly higher levels than LNCaP cells^27,29^, hence, the PSMA expression level of PC-3 PIP tumor xenografts does not exactly reflect the expression level of lesions in a patient.

### Therapy study and monitoring of mice

The therapy study was performed with 6 mice per group 7 days after inoculation of PC-3 PIP tumor cells (4 × 10^6^ cells, 100 μL Hank’s Balanced Salt Solution (HBSS)) on the right shoulder. At this stage, the tumors were still quite small (average ~60 mm^3^; Table 4) closely reflecting metastasized disease in patients with small lesions. At Day 0 of the study, animals of Group A were injected with 100 µL saline (NaCl solution 0.9%). Mice of Group B were injected with 6 MBq ^149^Tb-PSMA-617, mice of Group C were injected with 2 × 3 MBq ^149^Tb-PSMA-617 at Day 0 and at Day 1 and mice of Group D were injected with 2 × 3 MBq ^149^Tb-PSMA-617 at Day 0 and at Day 3. ^149^Tb-PSMA-617 was diluted to the respective activity with 100 μL saline (Table 4). The mice were monitored by measuring body weights and the tumor size every other day until the end of the study. Mice were euthanized when a predefined endpoint criterion was reached or when the study was terminated at Day 40. Endpoint criteria were defined as (i) body weight loss of >15%, (ii) a tumor volume of >800 mm^3^, (iii) a combination of body weight loss of >10% and a tumor volume of >700 mm^3^, (iv) signs of unease and pain or (v) a combination thereof. The relative body weight (RBW) was defined as [BW_x_/BW_0_], where BW_x_ is the body weight (in grams) at a given Day X and BW_0_ the body weight (in grams) at Day 0. The tumor dimensions were determined by measuring the longest tumor axis (L) and its perpendicular axis (W) with a digital caliper. The tumor volume (V) was calculated according to the equation [V = 0.5 * (L * W^2^)]. The relative tumor volume (RTV) was defined as [TV_x_/TV_0_], where TV_x_ was defined as the tumor volume in mm^3^ at a given Day X and TV_0_ the tumor volume in mm^3^ at Day 0. The anti-tumor efficacy of ^149^Tb-PSMA-617 was expressed as percentage tumor growth inhibition (% TGI), using the equation [(100 − (T/C)) × 100], where T is the mean RTV of treated mice and C is the mean RTV of control mice at the time of euthanasia of the first mouse of the control group. As an additional measure of the efficacy of the radionuclide therapy, the tumor growth delay indices were determined. The tumor growth delay (TGD_x_) was the time required for the tumor volume to increase x-fold over the initial volume at the Day 0. The tumor growth delay index [TGDI_x_ = TGD_x_(T)/TGD_x_(C)] was calculated as the TGD_x_ ratio of treated mice (T) over control mice (C) for a 2-fold (x = 2, TGD_2_) and 5-fold (x = 5, TGD_5_) increase of the initial tumor volume. The median lifetime, based on euthanasia of the mice when they reached an endpoint, was calculated using GraphPad Prism software (version 7). After euthanasia, kidneys, liver and the brain were collected and weighed. The organ ratios (kidney-to-brain and liver-to-brain) were calculated using the organ masses obtained at the day of euthanasia. Organ data were analyzed for significance using a one-way ANOVA test with a Tukey’s post correction (GraphPad Prism software, version 7). A p-value of <0.05 was considered as statistically significant.

**Table 4:** Design of the therapy study indicating the application scheme, as well as the average tumor volume and body weight of each group at therapy start.

Group	Treatment (n = 6)	Injected Radioactivity		Tumor Volume [mm^3^]	Body Weight [g]
		[MBq]	Days of Injections (Day)		
A	saline	—	—	46 ± 18	16 ± 0.6
B	^149^Tb-PSMA-617	1 × 6 MBq	Day 0	82 ± 56	17 ± 1.5
C	^149^Tb-PSMA-617	2 × 3 MBq	Day 0 and Day 1	56 ± 30	17 ± 0.7
D	^149^Tb-PSMA-617	2 × 3 MBq	Day 0 and Day 3	64 ± 15	17 ± 0.6

Values indicated as average ± SD.

Blood samples were taken at the time of euthanasia for the evaluation of a selection of clinical chemistry parameters of renal and hepatic function (creatinine, blood urea nitrogen, alkaline phosphatase, total bilirubin and albumin) (Supplementary information).

### PET/CT imaging studies

In a separate experiment, PET/CT scans were performed using a small-animal bench-top PET/CT scanner^40^ (G8, Perkin Elmer, Massachusetts, U.S), as previously reported, with a set energy window ranging from 150 keV to 650 keV^41^. Mice were subcutaneously inoculated with PC-3 PIP tumor cells (6 × 10^6^ cells) and PC-3 flu tumors cells (5 × 10^6^ cells) on the right and left shoulder, respectively, 7–10 days before the acquisition of the PET/CT scans. This mouse model with a PSMA-positive and a PSMA-negative tumor xenograft in one animal enables the determination of PSMA-specific radioligand uptake without the need for blocking studies using 2-(phosphonomethyl)-pentandioic acid (2-PMPA) as a PSMA inhibitor. During the scan, mice were anesthetized with a mixture of isoflurane and oxygen. Static whole-body PET scans of 10 min duration were performed at 30 min, 2 h and 4 h after injection of ^149^Tb-PSMA-617 (5 MBq, 1.2 nmol, 200 µL), followed by a CT scan of 1.5 minutes. The aquistion of the data and their reconstruction was performed using the G8 PET/CT scanner software (version 2.0.0.10). All images were prepared using *VivoQuant* post-processing software (version 3.5, inviCRO Imaging Services and Software, Boston U.S.). A Gaussian post-reconstruction filter (full width at half maximum = 1 mm) was applied to the images and the scale was adjusted by cutting 5–10% of the lower signal intensity to make the tumors and kidneys readily visible.

### Ethical approval

This study was performed in agreement with the national law and PSI-internal guidelines of radiation safety protection. *In vivo* experiments were approved by the local veterinarian department and conducted in accordance with the Swiss law of animal protection.

## Supplementary information

Supplementary Information
