# Supplementary material for: Alpha-PET for Prostate Cancer: Preclinical investigation using 149Tb-PSMA-617
Source: Sci Rep. 2019 Nov 28;9:17800. doi: 10.1038/s41598-019-54150-w (PMC6882876; doi:10.1038/s41598-019-54150-w)
Supplement: Supplementary file 1 — Supplementary Information [file 41598_2019_54150_MOESM1_ESM.pdf]

SUPPLEMENTARY INFORMATION

Alpha-PET for Prostate Cancer: Preclinical investigation using ^{149}Tb-PSMA-617

Christoph A. Umbricht[1], Ulli Köster[2], Peter Bernhardt[3,4], Nadezda Gracheva[1], Karl Johnston[5], Roger Schibli[1,6], Nicholas P. van der Meulen[1,7], Cristina Müller[1,6*]

1. Center for Radiopharmaceutical Sciences ETH-PSI-USZ, Paul Scherrer Institute, 5232 Villigen-PSI, Switzerland

2. Institut Laue-Langevin, 38042 Grenoble, France

3. Department of Radiation Physics, Institution of Clinical Science, Sahlgrenska Academy, University of Gothenburg, 413 45 Gothenburg, Sweden

4. Medical Bioengeneering, Sahlgrenska University Hospital, 413 45 Gothenburg, Sweden

5. CERN, 1211 Geneva 23, Switzerland

6. Department of Chemistry and Applied Biosciences, ETH Zurich, 8093 Zurich, Switzerland

7. Laboratory of Radiochemistry, Paul Scherrer Institute, 5232 Villigen-PSI, Switzerland

***Correspondence to**:
PD Dr. Cristina Müller
Center for Radiopharmaceutical Sciences ETH-PSI-USZ
Paul Scherrer Institute
5232 Villigen-PSI
Switzerland
e-mail: cristina.mueller@psi.ch
phone: +41-56-310 44 54; fax: +41-56-310 28 49

1. Quality Control of ^{149}Tb-PSMA-617

Experimental Procedure: A sample of ^{149}Tb-PSMA-617 was diluted in sodium diethylenetriamine pentaacetic acid (Na-DTPA; 50 µM) to perform quality control (QC) using HPLC (Merck Hitachi LaChrom L-7100 HPLC pump coupled with a L-7200 autosampler, a D-7000 interface and an HPLC radioactivity monitor LB 506 B from Berthold) equipped with a reversed-phase column (Xterra™, MS, C18, 5 µm, 150 x 4.6 mm; Waters). The mobile phase consisted of MilliQ water containing 0.1% trifluoroacetic acid (A) and acetonitrile (B). A gradient from 95% A and 5% B to 20% A and 80% B over a period of 15 min was used at a flow rate of 1 mL/min.

Results: the ^{149}Tb-PSMA-617 was obtained with a radiochemical purity of >98% up to a specific activity of 6 MBq/nmol (Figure S1).

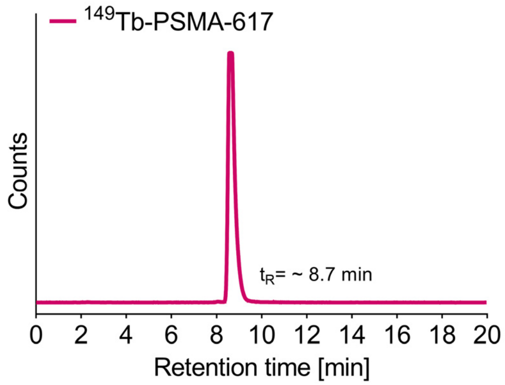

Figure S1. Representative HPLC chromatogram of ^{149}Tb-PSMA-617 eluted at a retention time of $t_R = 8.7$ min. Uncoordinated radiometal complexed with DTPA would appear at a retention time of $t_R = 2$ min, but was absent in this case.

2. Non-decay Corrected Biodistribution Data of ^{177}Lu-PSMA-617

Experimental Procedure: Biodistribution data of ^{177}Lu-PSMA-617 were previously published by Benešová et al.[1] These data were converted to non-decay-corrected values for the dose estimations of PC-3 PIP tumors, blood, kidneys and liver.

Results: The non-decay corrected biodistribution data of ^{177}Lu-PSMA-617 are shown in Table S1.

	**^{177}Lu-PSMA-617**			
**Time point p.i.**	**PC-3 PIP tumor n.d.c. [% IA/g]**	**Blood n.d.c. [% IA/g]**	**Kidneys n.d.c. [% IA/g]**	**Liver n.d.c. [% IA/g]**
15 min	32 ± 3.5	7.3 ± 0.3	31 ± 5	1.6 ± 0.3
30 min	36 ± 5	2.1 ± 0.2	17 ± 3	0.56 ± 0.03
1 h	44 ± 12	0.50 ± 0.06	9.7 ± 1.4	0.20 ± 0.04
2 h	45 ± 4	0.07± 0.01	3.9 ± 0.6	0.13 ± 0.02
4 h	55 ± 8	0.02 ± 0.00	3.6 ± 1.0	0.09 ± 0.01
6 h	41 ± 4	0.03 ± 0.00	2.0 ± 0.2	0.10 ± 0.00
24 h	34 ± 5	0.01 ± 0.00	0.68 ± 0.13	0.06 ± 0.01
48 h	23 ± 3	0.01 ± 0.00	0.29 ± 0.04	0.03 ± 0.00
96 h	14 ± 0.2	0.00 ± 0.00	0.13 ± 0.03	0.02 ± 0.01
192 h	8.6 ± 1.3	0.00 ± 0.00	0.07 ± 0.01	0.02 ± 0.00

Table S1. Non-decay-corrected values for ^{177}Lu-PSMA-617 based tissue distribution values previously published by Benešová et al.[1] Each data point represents the average of a group of mice ± SD (n = 3). Adapted with permission from (Benešová et al. 2018 Mol Pharm 15(3):934-946)[1]. Copyright (2019) American Chemical Society.

3. Calculated Biodistribution of ^{149}Tb-PSMA-617 and Respective AUCs

Theoretical Calculations: The previously-reported tissue distribution of ^{177}Lu-PSMA-617 revealed fast accumulation in PC-3 PIP tumor xenografts in mice, with the kidneys being the only normal organs with substantial accumulation of activity.[1] Transformation of these data to non-decay-corrected data, using the half-life of ^{149}Tb, revealed the time-dependent uptake of ^{149}Tb-PSMA-617 in the various tissue and enabled determination of the areas under the curves (AUCs) using the curve fit toolbox in MATLAB. The AUC ratios were calculated for the tumor uptake relative to the accumulation of the radioligand in the blood, kidneys and liver, respectively.

Non-decay-corrected values for ^{149}Tb-PSMA-617 were calculated based on the values measured for ^{177}Lu-PSMA-617, under the assumption that the tissue distribution of radiolabeled PSMA-617 would be equal, irrespective of the coordinated radiolanthanide.

Results: Non-decay-corrected biodistribution data of ^{149}Tb-PSMA-617, as well as the AUC values for the tumor and critical healthy organs and the respective AUC ratios, are listed in Table S2.

**A**	**Tissue Distribution of ^{149}Tb-PSMA-617**			
**Time point p.i.**	**PC-3 PIP tumor n.d.c. [% IA/g]**	**Blood n.d.c. [% IA/g]**	**Kidneys n.d.c. [% IA/g]**	**Liver n.d.c. [% IA/g]**
15 min	31 ± 4.3	7.0 ± 0.31	29 ± 4.3	1.6 ± 0.26
30 min	33 ± 4.8	2.0 ± 0.17	16 ± 2.8	0.52 ± 0.03
1 h	37 ± 10	0.42 ± 0.05	8.2 ± 1.2	0.17 ± 0.03
2 h	33 ± 2.8	0.05 ± 0.01	2.8 ± 0.4	0.10 ± 0.01
4 h	29 ± 4.1	0.01 ± 0.00	1.9 ± 0.53	0.05 ± 0.00
6 h	15 ± 1.6	0.01 ± 0.00	0.73 ± 0.06	0.04 ± 0.00
24 h	0.66 ± 0.10	0.00 ± 0.00	0.02 ± 0.00	0.00 ± 0.00
**B**	**AUCs of ^{149}Tb-PSMA-617**			
**Time period**	**Tumor**	**Blood**	**Kidneys**	**Liver**
0-∞h	58	4.9	34	1.6
**C**	**AUC ratios of ^{149}Tb-PSMA-617**			
**Time period**		**Tu-to-Bl**	**Tu-to-Ki**	**Tu-to-Li**
0-∞ h		74	10	225

Table S2. (**A**) Non-decay-corrected values for ^{149}Tb-PSMA-617 based on ^{177}Lu-PSMA-617 measurements published by Benešová et al.[1] Time-points later than 24 h p.i. are below 0.01% IA/g for all tissues and, therefore, not shown. (**B**) Area under the curve (AUC) values for selected organs and tissues obtained for data points of ^{149}Tb-PSMA-617 from 0 h to an infinite time p.i. (**C**) AUC ratios obtained based on AUC values of ^{149}Tb-PSMA-617; Each data point represents the average of values obtained from 3 mice.

4. Dosimetry Estimations for ^{177}Lu-PSMA-617

Experimental The mean specific absorbed doses (Gy/MBq) to the tumors and kidneys were calculated by multiplication of the time-integrated activity concentration, determined from created time-activity concentration curves, by the emitted electron energy of 147 keV for ^{177}Lu (www.nndc.bnl.gov), the absorbed electron fractions for the kidney and tumors (assessed by Monte Carlo simulations using PENELOPE-2014)[2] and a conversion factor. The time-activity concentration curve for the tumor and kidneys was achieved with a mono-exponential and bi-exponential function, respectively, fitted to the non-decay corrected data points. The time-integrated activity concentration was obtained by integration to infinity.

Results: The results of the dosimetry are given in the main article.

5. Blood Parameter Analysis after Therapy

Experimental Procedure: Blood was taken from the retrobulbar vein under isoflurane anesthesia immediately before euthanasia. Blood plasma parameters, such as alkaline phosphatase, blood urea nitrogen, creatinine, total bilirubin and albumin were assessed using a dry chemistry analyzer (DRI-CHEM 4000i, FUJIFILM, Japan) after centrifugation of the blood to obtain the plasma. The average blood plasma parameters of each group were analyzed for significance using a one-way ANOVA test with a Tukey's post correction (GraphPad Prism software, version 7). A *p*-value of <0.05 was considered as statistically significant.

Results: The values of the blood plasma parameters are shown in Table S3. There were no significant differences determined between the values of control mice and those of treated mice.

Group (n=6)	Alkaline phosphatase (U/L)	Blood urea nitrogen (mmol/L)	Creatinine (µmol/L)	Total bilirubin (µmol/L)	Albumin (g/L)
Group A	98 ± 66	8.0 ± 2.1	<18	4.8 ± 0.75	22 ± 2.3
Group B	102 ± 24	5.5 ± 1.7	<18	5.3 ± 1.63	22 ± 4.4
Group C	86 ± 51	5.4 ± 2.1	<18	5.2 ± 1.3	23 ± 2.0
Group D	111 ± 51	6.5 ± 1.9	<18	5.0 ± 1.0	22 ± 2.0

Values indicated as average ± SD

Data obtained at the day of euthanasia when an endpoint criterion was reached.

Table S3. Blood parameters of mice of the therapy study.

6. PET/CT Imaging Studies

Experimental Procedure: The experimental procedure of the PET/CT imaging studies is reported in the main article.

Results: Herein, we present the PET/CT images as sagittal, coronal and transaxial sections with increased CT contrast in order to make both tumors visible: the PSMA-expressing PC-3 PIP tumor xenograft on the right side of the shoulder and the PSMA-negative PC-3 flu tumor on the left side of the shoulder (Figure S2).

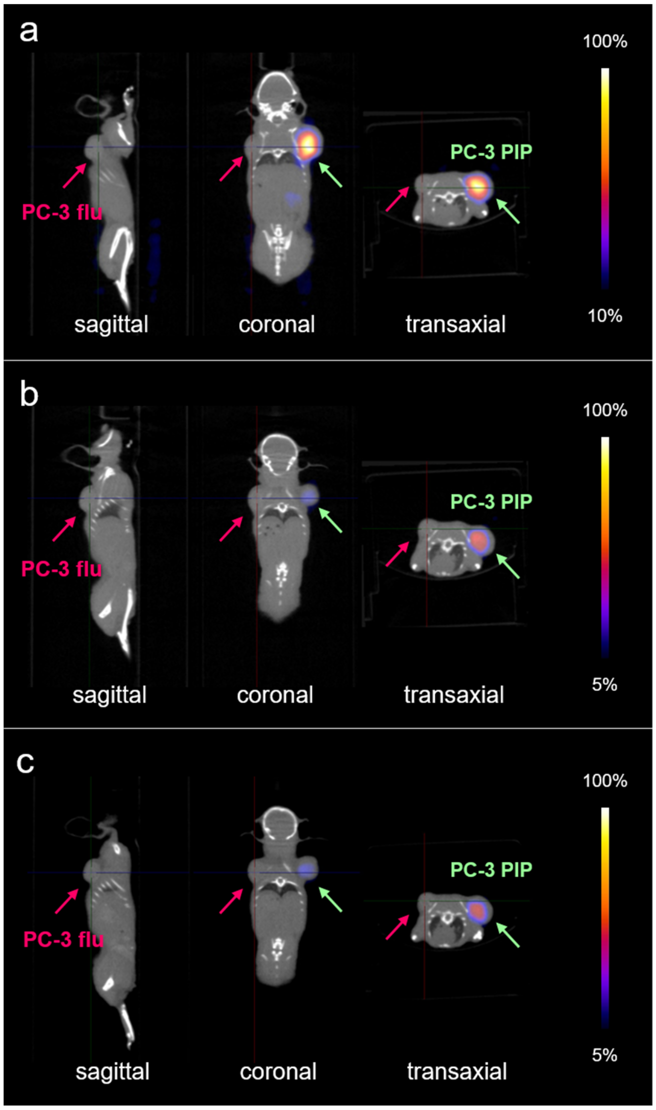

Figure S2. Sagittal, coronal and transaxial sections of PET/CT scans of a mouse bearing a PSMA-positive PC-3 PIP tumor xenograft (right shoulder) and PSMA-negative PC-3 flu (left shoulder) tumor xenografts. (**a**) PET/CT scan obtained 30 min after injection of ^{149}Tb-PSMA-617; (**b**) PET/CT scan obtained 2 h after injection of ^{149}Tb-PSMA-617; (**c**) PET/CT scan obtained 4 h after injection of ^{149}Tb-PSMA-617. PC-3 PIP = PSMA-positive tumor (indicated with green arrows); PC-3 flu = PSMA-negative tumor (indicated with red arrows).

References

1 Benešová, M., Umbricht, C. A., Schibli, R. & Müller, C. Albumin-binding PSMA ligands: optimization of the tissue distribution profile. *Mol Pharm* **15**, 934-946, doi:10.1021/acs.molpharmaceut.7b00877 (2018).

2 Salvat, F. PENELOPE-2014: A code system for monte carlo simulation of electron and photon transport. *OECD/NEA Data Bank NEA/NSC/DOC* **3**, http://www.nea.fr/lists/penelope.html (2015).
